# Effect of Constant Cyclic Stress Coupling on the Fatigue Behavior of 304LN Stainless Steel

**DOI:** 10.3390/ma17215220

**Published:** 2024-10-26

**Authors:** Huanchun Wu, Huiqiang Liu, Chaoliang Xu, Wenqing Jia, Qiwei Quan, Jian Yin, Yuanfei Li, Xiao Jin, Wangjie Qian, Haitao Dong, Xiangbing Liu

**Affiliations:** 1 Material Engineering Technology Center, Suzhou Nuclear Power Research Institute, Suzhou 215004, China; wuhuanchun1@163.com (H.W.);; 2 National Engineering Research Center for Nuclear Power Plant Safety & Reliability, Suzhou 215004, China; 3 Beijing Advanced Innovation Center for Materials Genome Engineering, Institute of Advanced Materials and Technology, University of Science and Technology Beijing, Beijing 100083, China

**Keywords:** coupled stress, crack propagation rate, crack tip, high-temperature water, corrosion fatigue

## Abstract

The stress state of the primary circuit main pipeline is very complex mixed constant stress and periodic cyclic stress during the operation of nuclear power plants, which often affects the failure behavior of the stainless steel (SS) pipe. The fatigue behavior of 304LN SS under mixed constant stress and cyclic stress was investigated, and it was found that the coupling effect of constant stress and cyclic stress has a significant collaborative acceleration on the fatigue behavior. The research results showed that the cracks in the 304LN SS did not propagate even after 78 days under both constant load conditions of 5 KN and 7.5 KN, while the crack growth rate (CGR) of the specimen increased by about an order of magnitude when coupled with a cyclic fatigue load. The fatigue life of 304LN SS was the longest under 200 °C high-temperature water, and its life was roughly the same under 250 °C and 300 °C water. In a 300 °C high-temperature water environment, the fatigue life under the coupling of constant stress and cyclic fatigue stress is significantly lower than that under symmetric cyclic stress alone. There is a synergistic acceleration effect on the fatigue life of 304LN SS when constant stress is coupled with periodic cyclic stress, which is attributed to the combined effect of a corrosion environment and mechanical factors.

## 1. Introduction

Austenitic stainless steel (SS) was widely used in the fields of energy, power, and chemical engineering due to its excellent mechanical and corrosion properties [1,2,3,4,5]. One of the typical ones commonly used as the primary piping system in nuclear power plants is 304LN SS, which often serves under stress and high-temperature and high-pressure water environments for a long time [6,7,8,9,10]. The stress state of the primary piping system during operation is extremely complex. On one hand, it experiences constant tensile stress due to the high-temperature and high-pressure coolant. On the other hand, it is subjected to alternating stress due to temperature and pressure variations of the coolant, as well as the reactor startup and shutdown cycles. Therefore, stress corrosion cracking (SCC) and corrosion fatigue are common failure modes for a main pipeline [11,12,13,14,15].

Currently, a number of studies have been conducted on SCC and corrosion fatigue of SSs in corrosion environments, leading to a deeper understanding of the relevant failure mechanisms. Was et al. [16] studied the SCCs of four materials (Alloy 304 SS, 316 SS, Inconel 625, and Inconel 690) in high-temperature water environments and discovered the relationship between crack initiation and the rupture of oxide film. It revealed the effect mechanism of oxidation on SCC under high-temperature water and steam environments. Meng and Du et al. [12,17] investigated the effect of different high-temperature water chemical environments on the SCC of SS and obtained the regularities of the effects of dissolved hydrogen (DH) and dissolved oxygen (DO) on SCC.

The corrosion fatigue of SS in high-temperature water is another research area. Wu et al. [18,19] studied several factors, such as sigma phase, thermal aging, and grain size on the corrosion fatigue life of SS in high-temperature water environments, clarifying the effect of materials on the service process of nuclear power piping materials. Zhao et al. [20] conducted corrosion fatigue tests on X80 pipeline low-alloy steel in a sodium chloride solution and found that corrosion products on the specimen surface would penetrate into the sub-surface, even the matrix, along with the movement of slip bands under cyclic tensile–compressive stress, thereby damaging the structural integrity of the metal sub-surface and leading to further crack initiation. The research results of Wu et al. [21] also indicated that Z3CN20.09M SS would experience the phenomenon of oxidation corrosion products penetrating into the material matrix and initiating cracks under fatigue stress in a high-temperature water environment. The effects of material, stress level, and water chemistry on SCC and corrosion fatigue have been studied widely, and the relevant effect patterns and failure mechanisms have been explored in depth [2,22,23,24,25,26,27].

However, the stress state of the primary piping system during service is often subjected to the coupling effect of constant stress and cyclic stress, as mentioned previously. The complex stress state leads to more complex failure modes [11,28,29], while there is still a lack of in-depth research on these failure modes. Lu et al. [3] studied the effect of different stress loading modes on 316NG SS and found that different stress loading types significantly affect the fracture mode of the material during crack propagation rate tests. When the loading stress changes from constant stress to dynamic stress, the crack propagation mode changes from transgranular to intergranular. Mannan et al. [30] made similar findings in their study on creep-fatigue tests of 316L SS.

During these studies, almost all researchers have only focused on the effect of different stress types on material fatigue behavior, while there is a lack of necessary attention and research on the coupling effect of constant stress and cyclic stress. In this study, it focuses on investigating the fatigue behavior under the coupling effect of constant stress and cyclic stress for 304LN SS, especially under a 300 °C high-temperature water environment. And it reveals the effect of the failure mode and mechanism induced by coupled stress based on a comparative analysis.

## 2. Materials and Methods

### 2.1. Material

**Figure 1:**
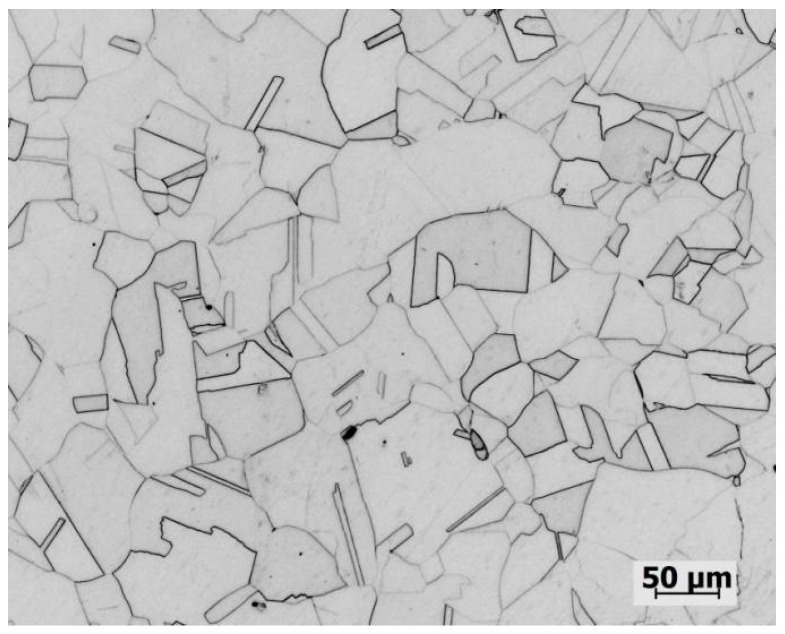
Metallographs of the 304LN SS.

The material used in this study is forged 304LN SS. The composition is shown in Table 1, and the microstructure is shown in Figure 1. The mechanical properties of the 304LN SS were as follows: elongation A% was 52%, yield strength R_p0.2_ was 290 MPa, and the tensile strength R_m_ was 620 MPa.

### 2.2. Specimen Design

**Figure 2:**
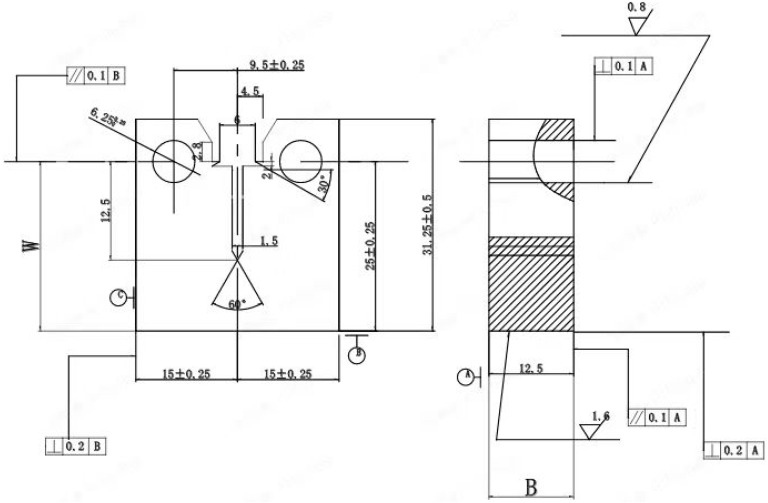

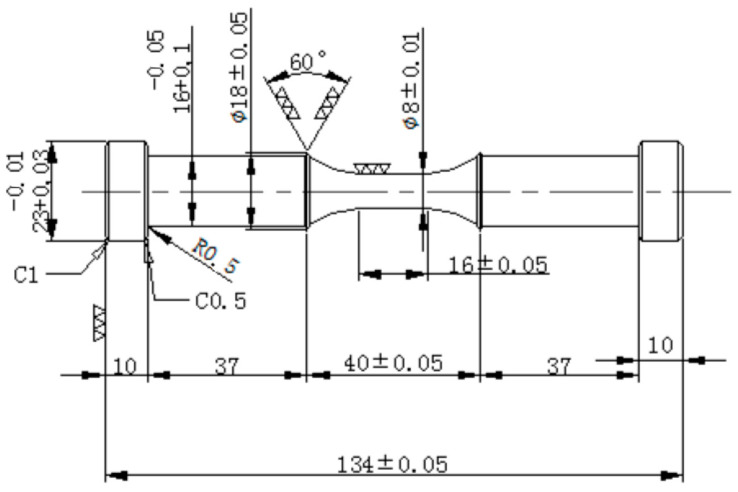
Compact-type specimen drawing used for the fatigue crack growth test (**up, B: Bold, W: Width**) and a rod-shaped specimen used for crack initiation process (**down**).

Figure 2 shows the specimens used in this study. Figure 2 (up) is a compact-type (CT) specimen used for the crack propagation test (1T). Figure 2 (down) shows the specimen used for the fatigue life test under a high-temperature water environment. The water chemistry and mechanical parameters of the corrosion fatigue test are listed in Table 2. After all the fatigue tests, the fracture surfaces of the specimens were observed using a scanning electron microscope (SEM, SUPRA55, ZEISS, Oberkochen, Germany).

### 2.3. Corrosion Fatigue Test

Before the corrosion fatigue test, the crack growth rate (CGR) test under constant load and coupled load was conducted at room temperature. The fatigue test was controlled by ΔK. The frequency and stress rate were 10 Hz and 0.1, respectively. The low-cycle fatigue tests were conducted in 200, 250, and 300 °C high-temperature water environments. All low-cycle fatigue tests were conducted in a high-temperature and high-pressure water-cracking system with a capacity dynamic load of 20 kN and a dynamic autoclave prepared by austenitic stainless steel. The instrument was operated by a computer-controlled servo-electric system. All the fatigue tests were conducted in the strain control mode with a triangular waveform. The strain was measured by an LVDT extensometer. The test parameters and high-temperature water chemistry are shown in Table 2. After the corrosion fatigue test, the fracture was observed by SEM.

## 3. Results and Discussion

### 3.1. Effect of Coupled Load on Crack Propagation Rate at Room Temperature 

In order to investigate the coupled effect of constant tensile stress and cyclic stress, crack propagation rate (CGR) tests were conducted on CT specimens under different stress types in a room temperature environment (about 25 °C). This preliminary study aimed to explore the fatigue behavior under coupled stresses.

#### 3.1.1. Crack Propagation under Constant Load

**Figure 3:**
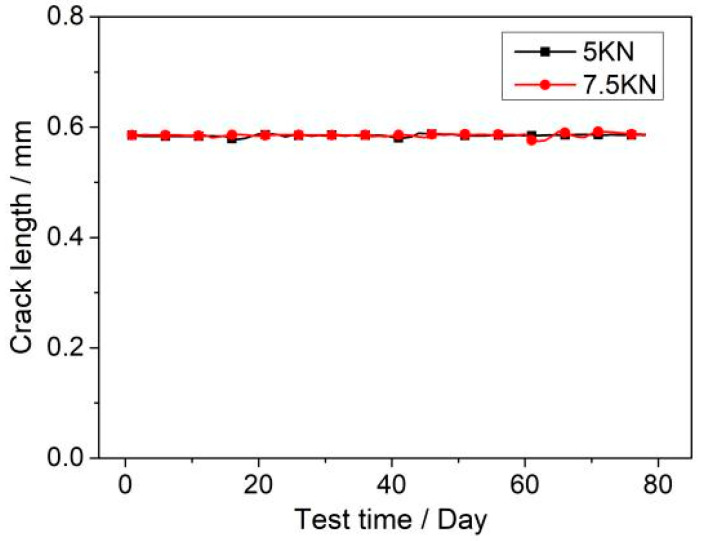
Crack length of 304LN SS under constant load conditions of 5 KN and 7.5 KN.

Figure 3 illustrates the variation of crack length for 304LN SS under constant tensile load conditions of 5 KN and 7.5 KN after approximately 78 days. It can be observed that the crack length in the CT specimen shows almost no change after 78 days, indicating that there is no significant crack propagation.

#### 3.1.2. Crack Propagation Rate Equation under Coupled Load

Based on the constant load test, a coupled load test was conducted with a constant tensile load and a periodic fatigue load. In the CGR test, the following two load levels were used: (1) a periodic cyclic load in the range of 0–10 kN, and (2) a periodic cyclic load in the range of 5–15 kN. The test was conducted using the method of pre-existing cracks, as follows. First, a 12.5 mm crack is prepared in the center of the CT specimen using wire cutting. Second, a 2 mm fatigue crack is prefabricated using fatigue equipment under the ΔK control mode. And then, the specimen was used for the fatigue test. The formula and calculation method for fitting the CGR are as follows.

The calculation of ΔK for the CT specimen, according to Equation (1), is:

(1) $$\Delta K=\frac{\Delta P}{B\sqrt{W}}\times \frac{\left(2+a\right)}{{\left(1-\alpha \right)}^{3/2}}(0.886+4.64\alpha -13.32\alpha^{2}+14.72\alpha^{3}-5.6\alpha^{4})$$

α=a/W in the equation.

(2) $$a/W=C_{0}+C_{1}U_{X}+C_{2}U_{x}^{2}+C_{3}U_{x}^{3}+C_{4}U_{x}^{4}+C_{5}U_{x}^{5}$$

(3) $$U_{X}={\left\{{\left[\frac{BEV_{x}}{P}\right]}^{1/2}+1\right\}}^{-1}$$

The corresponding coefficients are shown as Table 3.

The fitting formula for the fatigue CGR is:

(4) $$\frac{da}{dN}=C\Delta K^{n}$$

**Figure 4:**
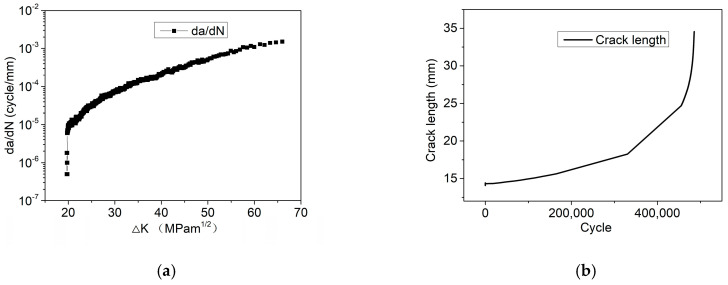
Crack propagation of 304LN SS under 0–10 kN cyclic fatigue load conditions: (**a**) CGR varies with the ∆K; (**b**) crack length varies with the fatigue life cycle.

Figure 4a shows the fitted curve of the relationship between the stress intensity factor and the CGR equation under a cyclic load in the range of 0–10 kN, as well as the crack length corresponding to the different cycle counts shown in Figure 4b. Then, the CGR equations were fitted using the methods mentioned above, as shown in Equation (5). Two of the same tests were conducted under the load condition of 0–10 kN. And the CGR equations fitted from the two results were similar, indicating the strong repeatability of the results.

(5) $$\frac{da}{dN}=3.56\times 10^{-11}\Delta K^{4.21}$$

**Figure 5:**
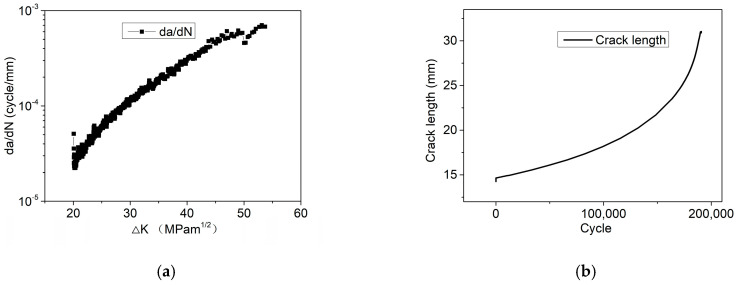
Crack propagation of 304LN SS under 5–15 kN cyclic fatigue load conditions: (**a**) CGR varies with the ∆K; (**b**) crack length varies with the fatigue life cycle.

Similarly, CGR tests were conducted under cyclic load in the range of 5–15 kN. The same method was used to fit the CGR equations, as shown in Equation (6). Figure 5a shows the fitted curve of the relationship between the stress intensity factor and the CGR equation, as well as the crack length corresponding to the different cycle counts shown in Figure 5b. Two of the same tests were conducted under the condition of 5–15 kN. And the CGR equations fitted from the two results were similar, indicating a strong repeatability of the results.

(6) $$\frac{da}{dN}=6.60\times 10^{-10}\Delta K^{3.54}$$

Comparing the CGRs under the two cyclic load conditions, it can be observed that the CGR is one order of magnitude under 5–15 kN higher than that under 0–10 kN, as indicated in Figure 4 and Figure 5. However, as mentioned above, no crack propagation occurs under a constant load of 5 kN and 7.5 kN after 78 days, indicating the CGR was zero. It can be inferred that the coupled effect of constant and cyclic load significantly increases the CGR for 304LN SS.

#### 3.1.3. Fracture Morphology under Different Load Conditions

**Figure 6:**
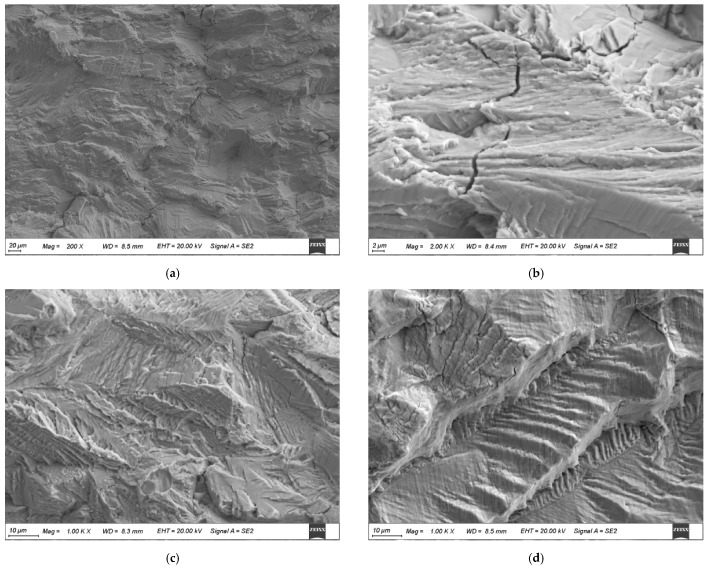
Fracture morphology of 304LN stainless steel under 0–10 kN cyclic fatigue load conditions: (**a**) early stage of crack propagation; (**b**) secondary cracks; (**c**) typical fatigue stripes; (**d**) twin grain crack.

**Figure 7:**
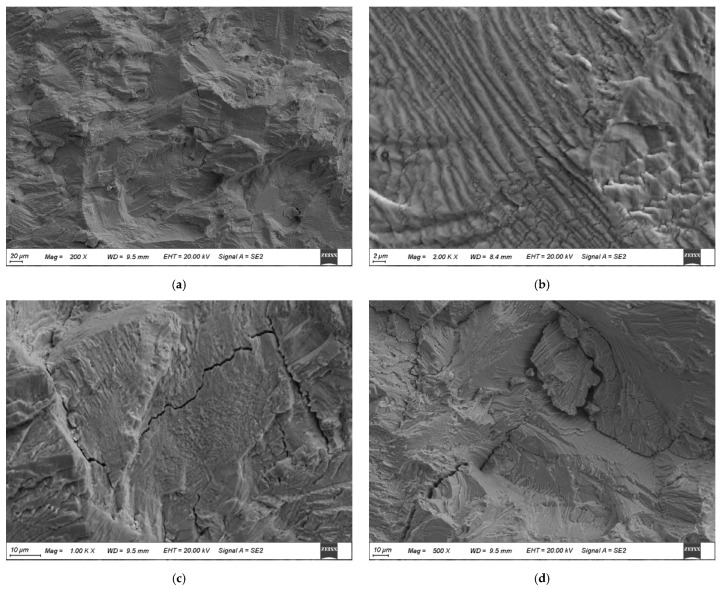
Fracture morphology of 304LN stainless steel under 5–15 kN cyclic fatigue load conditions: (**a**) early stage of crack propagation; (**b**) typical fatigue stripes; (**c**,**d**) secondary cracks.

Figure 6 and Figure 7 show the fracture surfaces of 304LN SS after CGR tests under different levels of fatigue load. From Figure 6, it can be observed that the fracture surface exhibits typical fatigue failure with several clusters of fatigue striations after the CGR test. The parallel fatigue striations can also form within the twinning during the fatigue test due to the low stacking fault energy with twin grains in the austenite phase, as shown in Figure 6d. In the fatigue fracture surface, secondary cracks can be observed in some areas, as shown in Figure 6b. Comparing the fracture surfaces under two different load levels, it is found that the overall morphology of the fracture surface is similar at different tensile loads, while there are more secondary cracks initiated and smaller spacing between fatigue striations in some areas at higher fatigue load, as shown in Figure 7. This indicates that the fatigue crack propagation of 304LN SS is faster under higher load conditions, leading to a more pronounced stress concentration near the crack tip and causing more secondary cracks.

According to the above test results, it can be concluded that the CGR of 304LN SS does not increase under the constant tensile loads of 5 KN and 7.5 KN, even over a period of 78 days. However, the CGR increases significantly under cyclic fatigue load, as the CGR equation is shown in Formula (5) with the load range of 0–10 kN. When the fatigue test was conducted under the cyclic fatigue load of 5–15 KN, as the constant tensile load of 5 KN and the cyclic fatigue load of 0–10 KN are combined, the CGR significantly increased as one order of magnitude higher than the 0–10 KN single fatigue load, as shown in Formula (6). The mathematical sum of the CGR for a constant load and a cyclic fatigue load is lower than it is under the combined effect of a fatigue load and a constant load, indicating a significant synergistic acceleration effect between the two load types. 

During the crack propagation process, the coupled complex load state often enhances the CGR. Numerous research results have shown a synergistic acceleration effect under the combined action of the constant load and cycle stress [11,28,29,30,31]. Maeng et al. [28] found that the CGR under the combined action of constant stress and cyclic stress in the study of Inconel 600 nickel-based alloy is much higher than the mathematical sum of CGR under constant stress and cyclic stress, indicating a synergistic acceleration effect. Lu et al. [29] studied the influence of stress mode on the crack propagation behavior of 316L SS and found that the CGR and fracture morphology of the material changed significantly under different loading modes. The cracking mode of the material changes from transgranular to intergranular, as observed by the fatigue fracture when using continuously changing dynamic stress loading. At the same time, the stress ratio has a significant effect on the CGR shown, as the larger the stress ratio the smaller the CGR. This indicates that the closer the cycle stress is to a single constant stress, the slower the CGR. Mannan et al. [30] also found in their study that the fatigue behavior of 316LN SS at a high temperature (500 °C) shows that the fatigue life decreases with the increase in tensile stress duration if the fatigue stress is sustained during the tensile process for a period of time. And the fracture mode changes from transgranular to a mixed mode of transgranular and intergranular. A further study found that the failure process of the material is significantly accelerated under the combined action of constant stress and cyclic stress. Through a comprehensive analysis of the above research results, it can be clearly seen that the combined action of constant stress and alternating stress can accelerate fatigue crack propagation. This study also confirmed the synergistic effect of tensile stress and periodic cyclic stress in high-temperature water environments, as shown in the following.

### 3.2. Effect of Coupled Stress on the Corrosion Fatigue of 304LN SS in 300 °C Water Environment

#### 3.2.1. Corrosion Fatigue Life under Coupled Stress

**Figure 8:**
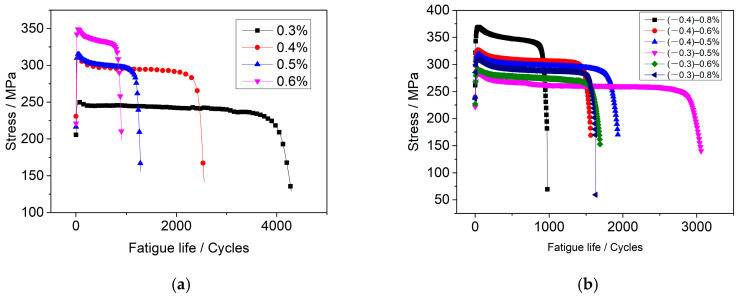
S-N curves of 304LN SS under different strain amplitudes in 300 °C high-temperature water environment: (**a**) symmetric stress, (**b**) asymmetric stress.

When cyclic stress is superimposed with constant stress, the effect of coupled stress on the corrosion fatigue of 304LN SS can be studied using asymmetric loading fatigue tests. Figure 8 shows the stress–life (S-N) curves of 304LN SS in a high-temperature water environment at 300 °C. Figure 8a represents the results of conventional symmetric stress tests with strain amplitudes of ±0.3%, ±0.4%, ±0.5%, and ±0.6%. One can see that the fatigue life significantly decreased with increasing the strain amplitude, as they were about 4000 and 1000 cycles for ±0.3% and ±0.6%, respectively. The peak fatigue stress shows a reversed trend, as it reached about 250 and 350 MPa for ±0.3% and ±0.6%, respectively. Figure 8b represents the results of corrosion fatigue tests under asymmetric stress with strain amplitudes ranging from (−0.4) to 0.8%, (−0.4) to 0.6%, (−0.4) to 0.5%, (−0.3) to 0.8%, (−0.3) to 0.6%, and (−0.3) to 0.5%. One can see that the fatigue life significantly decreased with increasing the tensile strain amplitude, as they were about 3000 and 1000 cycles for (−0.3)–0.5% and (−0.4)–0.8%, respectively. The peak fatigue stress shows a reversed trend, as it reached about 280 and 370 MPa for (−0.3)–0.5% and (−0.4)–0.8%, respectively. 

Comparing the results of the corrosion fatigue under symmetric stress and asymmetric stress, as mentioned above, it can be observed that the fatigue life of the specimen significantly decreased with increasing the tensile strain amplitude under the same compressive strain amplitude. This means that, when the tensile stress and symmetric fatigue stress are coupled, the fatigue life is significantly reduced. As indicated previously, the presence of constant tensile stress alone does not initiate a crack or promote crack propagation under the same environment. However, when it is superimposed with symmetric cyclic stress during fatigue testing, the fatigue life is significantly reduced. In other words, the sum of the results for constant tensile stress and cyclic stress separately cannot reach the combined effect as when they are applied coupled. Therefore, it can be inferred that constant stress and symmetric cyclic stress have an accelerated synergistic effect. 

#### 3.2.2. Corrosion Fatigue Fracture Morphology of Different Stress Types 

**Figure 9:**
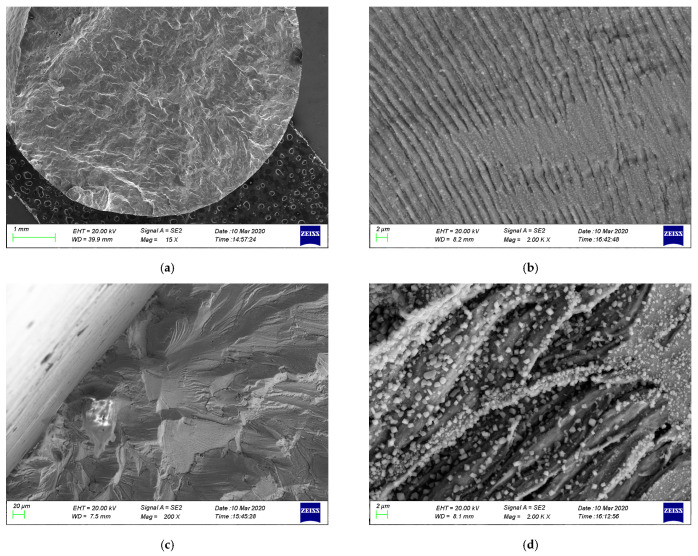
Fatigue fracture morphology of 304LN SS under symmetric stress at strain amplitude of ±0.3% in a 300 °C high-temperature water environment: (**a**) overall morphology of the fracture surface; (**b**) typical fatigue stripes; (**c**) crack initiation area; (**d**) oxides on the fracture surface.

**Figure 10:**
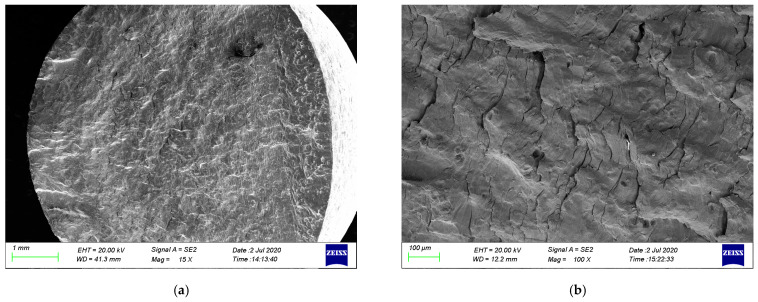

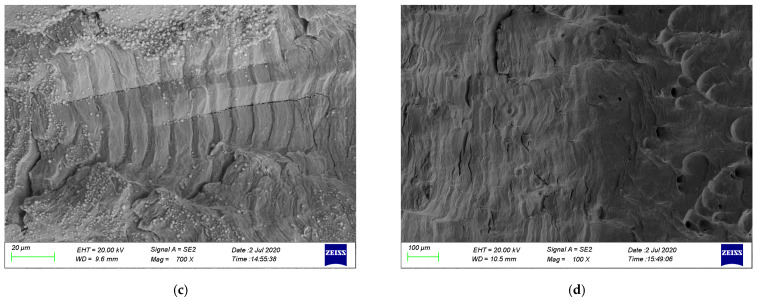
Fatigue fracture morphology of 304LN SS under cyclic stress and constant stress interaction at strain amplitude of (−0.3)–0.5% in 300 °C high-temperature water environment: (**a**) overall morphology of the fracture surface; (**b**) secondary cracks; (**c**) typical fatigue stripes; (**d**) crack propagation terminal area.

Figure 9 and Figure 10 show the fatigue fracture surfaces of 304LN SS in a 300 °C high-temperature water environment under symmetric stress and coupled stress, respectively. Comparing the fracture surfaces under the two stress conditions, it can be observed that the fatigue striation spacing on the fracture surface under symmetric stress is smaller, indicating a slower CGR. Additionally, more oxide particles can be observed on the fracture surface of the specimen under a symmetric stress test condition, and almost no secondary crack was observed. However, there are a lot of secondary cracks, and the crack tip shows a rapid propagation feature under coupled stress. This indicated that the CGR significantly increased under the coupling of constant stress and cyclic stress. Similar to fatigue tests at room temperature, the stress concentration around the fatigue crack is more severe under higher tensile stress conditions, leading to a faster CGR and easier initiation of secondary cracks.

In summary, the secondary crack is often easily initiated under higher fatigue stress or strain, both during the CGR and the fatigue life tests. The reasons can be attributed to the two aspects. On the one hand, the mechanical behavior under higher stress can induce crack initiation, as mentioned above. On the other hand, pay attention to the materials used in this study, namely 304LN SS with austenite phase. In this type of alloy, martensitic transformation is often observed under some load conditions, which induce loose ductility and become more brittle. This effect promotes crack propagation and also micro-crack initiation. In this study, this kind of effect cannot be denied, although no martensitic transformation was found.

**Figure 11:**
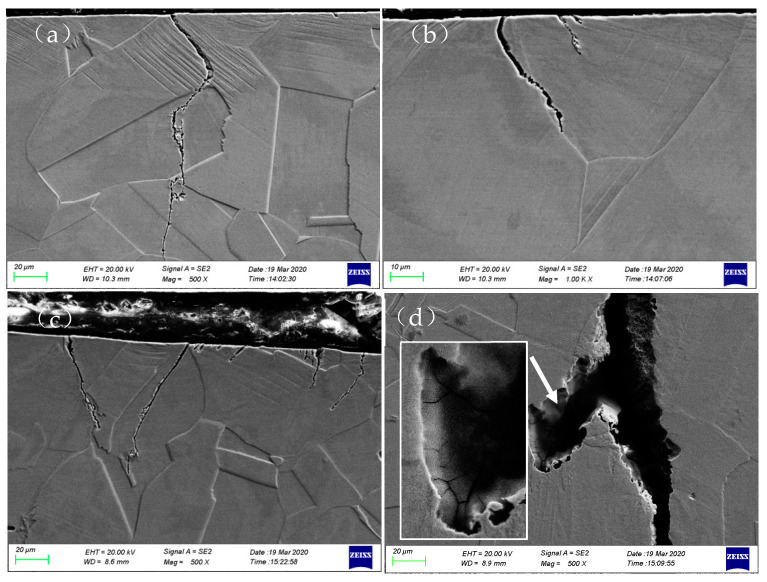
Morphology of the fracture cross-section of 304LN SS under different strain amplitudes under high-temperature water environment after fatigue fracture: (**a**,**b**): ±0.3%; (**c**,**d**): ±0.6%.

Figure 11 shows the cross-sectional morphology of the fracture surfaces of 304LN SS after fatigue failure under different strain amplitudes in a 300 °C high-temperature water environment. From the cross-sectional views of the specimens, it can be observed that fatigue cracks generally initiate at the persistent slip bands (PSBs) or grain boundaries. Figure 11a,b shows the typical morphologies of crack initiation at the PSBs and grain boundaries, respectively. Comparing the crack initiation under different strain amplitudes, it is easier for small cracks to initiate at the surface of the specimens under higher strain amplitudes, as shown in Figure 11c. Additionally, secondary cracks can also initiate within the main crack formed under higher strain amplitudes, as shown in Figure 11d. In summary, a fatigue crack in 304LN SS can be initiated transgranularly or intergranularly in a high-temperature water environment, generally at the PSBs or grain boundaries. Moreover, the secondary crack initiation increased with increasing the fatigue strain amplitude.

Based on the research results mentioned above, it can be concluded that constant stress may not have a significant effect on crack propagation when applied separately from cyclic periodic stress. However, it has a significant effect on the fatigue life of the specimen when coupled with cyclic stress, which indicates the presence of a coupling synergistic acceleration effect [23,24]. The reasons for this coupling acceleration effect can be attributed to both mechanical and corrosion factors. First, the increase in constant tensile stress leads to an increase in the stress concentration on the material surface and crack tip during the fatigue stress cycle, resulting in increased plastic deformation and crack initiation in localized areas.

Second, the fresh metal on the surface was pulled out of the material matrix and oxidized by the high-temperature water when the fatigue specimen was subjected to a suitable tensile stress. When compressive stress was applied, the oxides were pushed into the matrix along with the slip bands. After a certain number of cycles, the matrix of the metal was damaged by the combined action of slip bands and oxides, leading to a fatigue crack finally being initiated. In fact, the effect of tensile stress on crack initiation was magnified by the movement of the slip band and oxide [30]. Under normal symmetric stress conditions, there is limited exposure of the fresh metal when tensile stress is applied, resulting in limited oxides forming in high-temperature water. However, when constant tensile stress, coupled with symmetric stress, is applied, the higher tensile stress results in more fresh metal being pulled out by the slip bands and more oxides being formed. When compressive stress was applied, more oxides penetrated into the material matrix, causing more severe damage to the matrix metal. Consequently, crack initiation occurs in a shorter time and leads to a shorter fatigue life.

### 3.3. Effect of Temperature on the Corrosion Fatigue of 304LN SS in High-Temperature Water Environment

#### 3.3.1. Corrosion Fatigue Life under Different High-Temperature Water Environments

**Figure 12:**
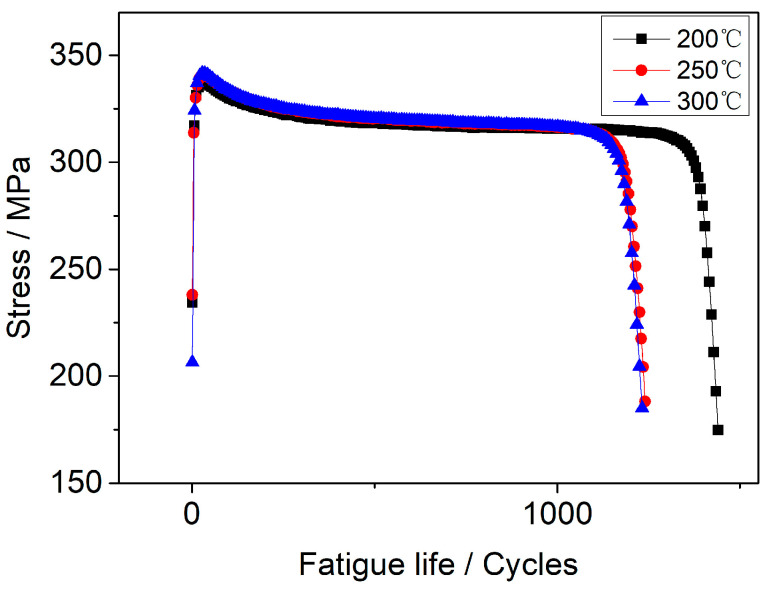
S-N curves of 304LN SS under different temperature water environments at strain amplitude of ±0.6%.

Figure 12 shows S-N curves of 304LN SS under different temperature water environments at strain amplitude of 0.6%. One can see that the fatigue life was roughly the same under 250 °C and 300 °C in a high-temperature water environment. The fatigue life of 304LN SS was the longest under 200 °C high-temperature water than the same environment under 250 °C and 300 °C by about 15%. The peak fatigue stress was almost the same at three different test temperatures.

The effect of temperature on the fatigue performance of metal materials is complex [32,33,34]. Generally speaking, the fatigue life increases with the increasing temperature below 150 °C, while the effect is relatively small. The effect of temperature on the fatigue life becomes obvious with the test temperature further increased, reaching a peak between 175–250 °C for most metals. However, as the temperature continues to rise to 320 °C, the effect of temperature on the fatigue life will be decreased. Li et al. [33] found that the temperature of the corrosion fatigue behavior of Alloy 690TT steam generator (SG) tubes was very obvious from 25 to 325 °C. Their study result shows that temperature can affect the crack initiation mechanism in persistent slip bands (PSBs), as well as the crack propagation mode. And it led to the corrosion fatigue life of Alloy 690TT decreasing with the increase in water temperature. Furthermore, Ghazi et al. [32] found that the fatigue crack growth rate (FCGR) of the Inclonel 625 alloy was affected by temperature, as it changes metal ions activation energy on the specimen surface and diffusion coefficients. Moreover, mechanical factors play the one of leading factors in temperature rise from 550 to 650 °C in a supercritical water (SCW) steam environment. The effect of temperature on the fatigue behavior of 316LN SS in 100–320 °C high-temperature water was studied by Zhang et al. [34]. It is found that the fatigue life decreases with an increasing temperature in a high-temperature water environment. The fatigue cracks tended to nucleate at the PSBs and propagate under the shear stress, mainly due to the mechanical damage in lower-temperature water, while the fatigue crack tended to propagate perpendicular to the loading direction due to severe electrochemical corrosion effects in higher temperatures.

In a word, the effect of the temperature on the fatigue life of the metal alloy was complex, especially in corrosion environments such as high-temperature water or SCW steam. This is attributed to the oxidation process, which can destroy the integrity of the material matrix under the movement of the PSBs, and this process could be enhanced with the increase in water temperature. Consequently, the fatigue performance was often mainly dependent upon the mechanical loading factor under a relatively low temperature, while it depended on the water environment factor under a relatively high temperature. In the present study, the fatigue life of 304LN SS was decreased with increasing the temperature under 300 °C, and the effect of temperature was small from 250 to 300 °C.

#### 3.3.2. Corrosion Fatigue Fracture Morphology of Different Temperature

**Figure 13:**
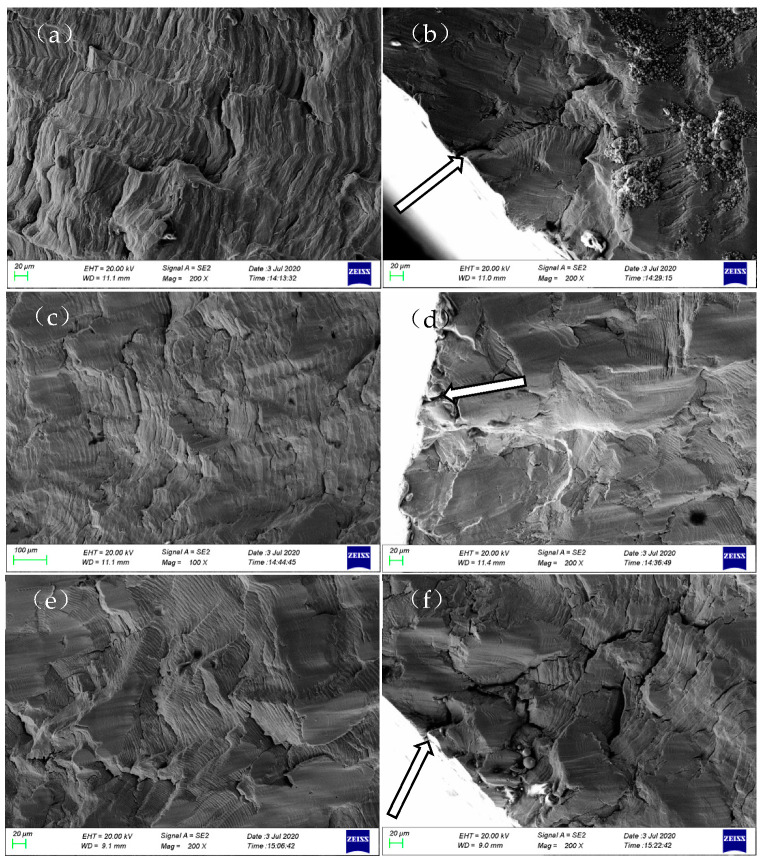
Fatigue fracture morphology of 304LN SS under different high-temperature water environments at strain amplitude of ±0.6%: (**a**,**b**) 200 °C; (**c**,**d**) 250 °C; (**e**,**f**) 300 °C.

Figure 13 shows the fracture morphology of 304LN SS under different-temperature water environments at a strain amplitude of ±0.6%. One can see that the fatigue crack is often initiated at the surface of the specimen, as indicated by the arrow. On one hand, the PSBs would prioritize forming on the surface and sub-surface of the fatigue specimen due to the special stress state on the surface. On the other hand, the specimen surface and the fresh metal exposed will be oxidized and penetrated into the specimen matrix along with the movement of PSBs during fatigue loading in a high-temperature water environment. The combination of two factors leads to the initiation of a fatigue crack on the surface and sub-surface of the specimen. Furthermore, the more the corrosion product penetrates into the matrix metal under the higher-temperature water finally results in faster crack initiation and shorter fatigue life.

It also can be seen that more second cracks were initiated on the fatigue fracture under higher temperature water, as indicated in Figure 13f. The higher the temperature, the more active the metal atoms. And they are more prone to corrosion and oxidation. It can be inferred that the formation of secondary cracks on the fatigue fracture surface is closely related to the entry of high-temperature water into the interior of the crack tip after the initiation of surface cracks. As mentioned previously, the surface crack initiates faster under the higher temperature, leading to more high-temperature water entering the crack tip. So, the more severe corrosion and oxidation occurred with more oxides penetrating into the fracture surface. Therefore, more secondary cracks were initiated on the fracture surface with the higher temperature. Although the fatigue life was similar at 250 and 300 °C, the more severe corrosion and oxidation led to more second cracks being initiated on the fatigue fracture in a 300 °C water environment.

## 4. Conclusions

In the present study, the effect of constant cyclic stress coupling on the fatigue behavior of 304LN SS was investigated. Based on the experimental results, the main conclusions are as follows.

(1) The cracks did not propagate even after 78 days under constant loads of 5 KN and 7.5 KN for 304LN SS at room temperature. The fatigue CGR of the specimen increased by about an order of magnitude when coupled with the constant load cyclic fatigue load induced by a synergistic acceleration effect;
(2) The fatigue life was the longest under 200 °C high-temperature water for 304LN SS, and its fatigue life was roughly the same under 250 °C and 300 °C water. The peak fatigue stress was almost the same at three different test temperatures;
(3) In a 300 °C high-temperature water environment, the fatigue life under the coupling of constant stress and cyclic symmetric fatigue stress is significantly lower than that under cyclic symmetric stress lonely. The fracture surface shows an increase in fatigue striation spacing under higher tensile stress, and the number of secondary cracks increased significantly;
(4) The synergistic acceleration effect of coupled stress was attributed to the fact that the higher tensile stress accelerated the plastic deformation from the slip band and formed more oxides in a high-temperature water environment. This leads to damage to the matrix during the movement of the slip bands, further promoting the fatigue crack initiation and propagation.

## Figures and Tables

**Table 1:** Chemical composition of 304LN SS (wt.%).

Element	C	Si	Mn	P	S	Cr	Ni	Mo	N	Fe
304LN	0.015	0.36	0.81	0.017	0.002	18.23	9.27	0.21	0.13	Balance

**Table 2:** Test parameters and high-temperature water chemistry for corrosion fatigue experiments.

Wave Form	Strain Amplitude	Strain Rate	Dissolved Oxygen	pH	Conductivity	Temperature	Pressure
Triangular	±0.3~±0.8%	0.001/s	10 ppm	6.6	0.15 µS/cm	200, 250, 300 °C	8 MPa

**Table 3:** Corresponding coefficients in equation 2.

Location	X/W	C0	C1	C2	C3	C4	C5
V0	−0.250	1.0010	−4.6695	18.460	−236.82	1214.8	−2143.6

## Data Availability

The raw data supporting the conclusions of this article will be made available by the authors on request.

## References

[1] Wu H., Yang B., Wang S., Zhang M., Shi Y., Chen Y. (2016). Effect of thermal aging on corrosion fatigue of Z3CN20.09M duplex stainless steel in high temperature water. Mater. Sci. Eng. A.

[2] Seifert H., Ritter S., Leber H. (2012). Corrosion fatigue crack growth behaviour of austenitic stainless steels under light water reactor conditions. Corr. Sci..

[3] Lu Z., Shoji T., Meng F., Qiu Y., Dan T., Xue H. (2011). Effects of water chemistry and loading conditions on stress corrosion cracking of cold-rolled 316NG stainless steel in high temperature water. Corr. Sci..

[4] Ti W., Wu H., Xue F., Zhang G., Peng Q., Fang K. (2021). Effect of thermal aging on the mechanical, intergranular corrosion and corrosion fatigue properties of Z3CN20.09M cast duplex stainless steel. Nucl. Eng. Technol..

[5] Ritter S., Seifert H. (2008). Effect of corrosion potential on the corrosion fatigue crack growth behaviour of low-alloy steels in high-temperature water. J. Nucl. Mater..

[6] Xia D., Sun Y., Fan H. (2015). Characterization of passive film formed on 304 SS in simulated alkaline water chemistries containing sulfur at 300 ℃. Tran. T.J. Univ..

[7] Liao J., Tan J., Wu X., Ning D., Xue G., Yao W. (2019). Corrosion fatigue behavior of 304 stainless steel notched specimen in high- temperature pressurized water. Mater. Sci. Eng. A.

[8] Cissé S., Laffont L., Tanguy B., Lafont M., Andrieu E. (2012). Effect of surface preparation on the corrosion of austenitic stainless steel 304L in high temperature steam and simulated PWR primary water. Corr. Sci..

[9] Chang L., Volpe L., Wang Y., Burke M., Maurotto A., Tice D. (2019). Effect of machining on stress corrosion crack initiation in warm forged type 304L stainless steel in high temperature water. Acta Mater..

[10] Yeh T., Huang G., Wang M., Tsai C. (2013). Stress corrosion cracking in dissimilar metal welds with 304L stainless steel and Alloy 82 in high temperature water. Prog. Nucl. Energ..

[11] Yaguchi S., Yonezawa T. (2014). Intergranular Stress Corrosion Cracking growth perpendicular to fatigue pre-cracks in T–L oriented compact tension specimens in simulated Pressurized Water Reactor primary water. Corr. Sci..

[12] Du D., Wang J., Chen K., Zhang L., Andresen P. (2019). Environmentally assisted cracking of forged 316LN stainless steel and its weld in high temperature water. Corr. Sci..

[13] Meisnar M., Vilalta-Clemente A., Moody M., Arioka K., Lozano-Perez S. (2016). A mechanistic study of the temperature dependence of the stress corrosion crack growth rate in SUS316 stainless steels exposed to PWR primary water. Acta Mater..

[14] Zhang W., Fang K., Hu Y., Wang S., Wang X. (2016). Effect of machining-induced surface residual stress on initiation of stress corrosion cracking in 316 austenitic stainless steel. Corr. Sci..

[15] Du D., Chen K., Lu H., Zhang L., Shi X., Xu X. (2016). Effects of chloride and oxygen on stress corrosion cracking of cold worked 316/316L austenitic stainless steel in high temperature water. Corr. Sci..

[16] Was G., Ampornrat P., Gupta G., Teysseyre S., West E., Allen T. (2007). Corrosion and stress corrosion cracking in supercritical water. J. Nucl. Mater..

[17] Meng F., Lu Z., Shoji T., Wang J., Han E., Ke W. (2011). Stress corrosion cracking of uni-directionally cold worked 316NG stainless steel in simulated PWR primary water with various dissolved hydrogen concentrations. Corr. Sci..

[18] Wu H., Li C., Fang K., Xue F., Yang B., Song X. (2017). Effect of grain size on the low cycle fatigue behavior of 316LN stainless steel in high temperature water. Mater. Corr..

[19] Wu H., Yang B., Wang Y. (2015). Effect of sigma phase on the low cycle fatigue property of Z3CN20.09M cast duplex stainless steel in high temperature water. Mater. Corr..

[20] Zhao W., Wang Y., Zhang T., Wang Y. (2012). Study on the mechanism of high-cycle corrosion fatigue crack initiation in X80 steel. Corr. Sci..

[21] Wu H., Yang B., Shi Y., Gao Q., Wang Y. (2015). Crack Initiation Mechanism of Z3CN20.09M Duplex Stainless Steel during Corrosion Fatigue in Water and Air at 290 °C. J. Mater. Sci. Technol..

[22] Ehrnstén U., Andresen P., Que Z. (2024). A review of stress corrosion cracking of austenitic stainless steels in PWR primary water. J. Nucl. Mater..

[23] Mukahiwaa K., Sceninia F., Burkea M., Plattsb N., Ticeb D., Stairmandb J. (2018). Corrosion fatigue and microstructural characterisation of Type 316 austenitic stainless steels tested in PWR primary water. Corr. Sci..

[24] Seifert H., Ritter S. (2008). Corrosion fatigue crack growth behaviour of low-alloy reactor pressure vessel steels under boiling water reactor conditions. Corr. Sci..

[25] Seifert H., Ritter S., Leber H. (2012). Corrosion fatigue initiation and short crack growth behaviour of austenitic stainless steels under light water reactor conditions. Corr. Sci..

[26] Duff J., Marrow T. (2013). In situ observation of short fatigue crack propagation in oxygenated water at elevated temperature and pressure. Corr. Sci..

[27] Cho H., Kim B., Kim I., Jang C. (2008). Low cycle fatigue behaviors of type 316LN austenitic stainless steel in 310 °C deaerated water–fatigue life and dislocation structure development. Mater. Sci. Eng. A.

[28] Maeng W., Kang Y., Nam T., Ohashi S., Ishihara T. (1999). Synergistic interaction of fatigue and stress corrosion on the corrosion fatigue crack growth behavior in Alloy 600 in high temperature and high pressure water. J. Nucl. Mater..

[29] Lu Z., Shoji T., Takeda Y., Ito Y., Kai A., Yamazaki S. (2008). Transient and steady state crack growth kinetics for stress corrosion cracking of a cold worked 316L stainless steel in oxygenated pure water at different temperatures. Corr. Sci..

[30] Mannan S., Valsan M. (2006). High-temperature low cycle fatigue, creep–fatigue and thermomechanical fatigue of steels and their welds. Int. J. Mech. Sci..

[31] Nam S. (2002). Assessment of damage and life prediction of austenitic stainless steel under high temperature creep–fatigue interaction condition. Mater. Sci. Eng. A.

[32] Ghazi A., Khan H., Farooq M., Jahangir S., Anwar M. (2023). Effect of temperature and medium environment on corrosion fatigue behavior of Inconel 625. Mater. Corr..

[33] Li Z., Lu Y., You L., Zhang X., Wang L., Tian D. (2024). Effect of temperature on corrosion fatigue behavior of Inconel Alloy 690TT steam generator tube. Corr. Sci..

[34] Zhang Z., Tan J., Wu X., Han E., Ke W., Rao J. (2019). Effects of temperature on corrosion fatigue behavior of 316LN stainless steel in high-temperature pressurized water. Corr. Sci..

